# Relationship between long-term use of proton pump inhibitor (PPI) and hypomagnesemia in patients with gastroesophageal reflux disease

**DOI:** 10.22088/cjim.13.1.107

**Published:** 2022

**Authors:** Abbas Arj, Zeinab ghaleh Takizadeh, Hamireza Gilassi, Mohsen Razavizadeh

**Affiliations:** 1 Department of Internal medicine, School of Medicine, Shahid Beheshti Hospital, Kashan University of Medical Sciences, Kashan, Iran; 2 Department of Epidemiology and Biostatics, Health Faculty, Kashan University of Medical Sciences, Kashan, Iran

**Keywords:** Gastroesophageal reflux disease, Hypomagnesemia, Proton pump inhibitor

## Abstract

**Background:**

The aim of this study was to evaluate the relationship between the long-term use of PPI and hypomagnesemia in patients with gastroesophageal reflux disease.

**Methods:**

This case control study was conducted on GERD patients with long-term use of proton pump inhibitor and patients with no history of gastroesophageal reflux and proton pump inhibitor referring to gastrointestinal clinic in 2019. Then concentration of serum magnesium (Mg) and potassium (K) were measured using atomic absorption spectrophotometer according to protocol. Other data were extracted from medical records. Statistical tests such as t-test, chi-square test and ONE WAY ANOVA were used for analysis of data.

**Results:**

In the current study, 263 patients were classified into two groups (case: 132, control: 131). The mean level of potassium in case and control groups was 3.92±0.64 and 4.20±0.43, respectively (P=0.001). Moreover, the mean level of Mg in two groups was 2.03±0.36 and 2.09±0.52, respectively (P=0.24). In addition, significant difference was seen between serum level of K, regarding the type of proton pump inhibitor and duration of medication use (p<0.01). However, no significant difference was seen between serum levels of Mg, regarding the type of proton pump inhibitor such as *omeprazole, *pantoprazole and other drugs (p>0.05).

**Conclusion:**

Based on these results, long-term use of proton pump inhibitors is not associated with hypomagnesemia in GERD patients. However, long-term use of PPIs may reduce serum potassium levels in these patients. Therefore, periodic evaluation of serum Mg level in PPI-treated patients seems to be unnecessary.

Recently, prevalence of gastroesophageal reflux disease (GERD) has increased in the world including Iran (1, 2). One of the significant effects of GERD is increasing the occurrence of adenocarcinoma of distal esophagus which is directly correlated with GERD (1, 3-7). Risk factors of GERD are environmental factors including obesity, consumption of various foods and genetic factors (1). Proton pump inhibitors (PPIs) are considered as the main basis of treatment for acid-related illnesses, such as GERD, peptic ulcer disease, and functional dyspepsia (8-11). They are one of the most prescribed classes of drugs in primary and specialty care (12). The Food and Drug Administration identified PPIs as effective and safety drugs in 2011 (13, 14), *strengthening the importance of *long term use of PPIs prescription in this association (8). However, the use of PPI may lead to some adverse events, such as interstitial nephritis (15), clostridium difficile colitis (16), respiratory infections (17), and hip fractures (18).

According to findings, PPIs may induce hypomagnesemia in patients. The association between the use of PPI and symptomatic hypomagnesemia was first described in two patients in 2006 (19). After this report, many reports have supported the association between the use of PPI and induced hypomagnesemia (20-24). The mechanism of PPI-associated hypomagnesemia is related to altered intestinal absorption of magnesium with long-term PPI use (13). In addition, low level of magnesium or hypomagnesemia is associated with variety of adverse events (AEs), such as vomiting, diarrhea, cramps, and even death (25, 26). In addition, hyperkalemia as an adverse effect of PPI therapy was observed in some studies (27, 28); however, another study reported increase in the frequency of hypokalemia after receiving PPIs use (29). 

Considering the importance of gastroesophageal reflux disease, the adverse effect of hypomagnesemia was induced by PPI and few and controversial studies regarding long-term use of proton pump inhibitor (PPI) and hypomagnesemia (30, 31). The aim of this study was to evaluate the relationship between the long-term use of proton pump inhibitor (PPI) and hypomagnesemia in patients with gastroesophageal reflux disease. In addition, the relation between the use of PPI and potassium level has been evaluated in these patients

## Methods

This case control study was conducted on GERD patients with long-term use of proton pump inhibitor (at least 1 year) and patients without history of gastroesophageal reflux and proton pump inhibitor referring to gastrointestinal clinic of Shahid Beheshti hospital in Kashan during 2019. The number of patients was assessed according to formula 1 and obtained 132 cases in each group (*95* % *confidence* interval and *80* % *power).*

$n=\frac{{\left(S_{1}^{2}+S_{2}^{2})(Z_{1-\frac{a}{2}}+Z_{1-\beta }\right)}^{2}}{{\left({\overset{\text{®}}{x}}_{1}-{\overset{\text{®}}{x}}_{2}\right)}^{2}}$

 After taking consent from patients, current study was approved by Ethics Committee of Kashan University of Medical Sciences. Inclusion criteria were GERD patients with long-term use of proton pump inhibitor. Exclusion criteria were hypokalemia, symptoms of malabsorption, kidney diseases, endocrine diseases, patient dissatisfaction and the use of diuretics. Data including age, gender, dosage and duration of PPI use were extracted from medical records. Moreover, questions were also asked about the patients' diets. Two groups were matched in terms of underlying variables. After collecting blood from patients, serum was separated by centrifuge (Eppendorf) and concentration of magnesium (Mg) and potassium (K) were measured using atomic absorption spectrophotometer (variant 20 plus) according to varian protocol in the laboratory of Shahid Beheshti Hospital. In the next step, the level of magnesium and potassium was compared in two groups. 

**Statistical analysis: **All data were entered to SPSS, Version 16. Statistical tests such as t- test, chi-square test and one way ANOVA were used for data analysis. A p-value < 0.05 was considered statistically significant. 

## Results

In the current study, 263 GERD patients with long-term use of proton pump inhibitor and patients with no history of gastroesophageal reflux were classified into two groups (case: 132, control: 131). The mean age of patients in case and control was 47.7±11.9 and 55.95±18.8 years, respectively (P=0.001). The duration of medication use was 3.01±2.7 years. Table 1 shows the frequency of variables including gender, proton pump inhibitor type, diuretic intake, and history of disease in case and control groups. Table 2 shows the comparison of case and control groups, regarding serum level of potassium and magnesium. 

**Table 1:** Frequency of variables including gender, proton pump inhibitor type, diuretic intake, and history of disease in case and control groups

**Parameters**	**Case**	**Control**	**Total**
**Gender**			
Male Female	46(34.7) 86(65.2)	65(49.6) 66(50.4)	111(41.8) 152(58.2)
**Proton** **pump** **inhibitor** **type**			
*Omeprazole* Pantoprazole Other No medication	92(69.7) 34(25.8) 6 (4.5) 0 (0)	0 ( 0) 0 (0) 0 (0) 131(100)	92(34.9) 34(25.8) 6 (2.2) 131(50)
**Diuretic intake**			
Yes No	0 (0) 132(100)	0 (0) 131(100)	0 (0) 263(100)
**History of disease**			
Mal absorption disease kidney disease Glandular disease	0 0 0	0 0 0	0 0 0

**Table 2:** Comparison of case and control groups, regarding serum level of potassium and magnesium

**Parameters**	**Case**	**Control**	**p-value**
Potassium (mEq/L)	3.92± 0.64	4.20±0.43	0.001
Magnesium (mg/ dl)	2.03± 0.36	2.09± 0.52	0.24

* Independent T test

As shown in table 2, there was a significant difference between case and control groups, regarding serum level of potassium (p<0.01). But no significant difference was observed between case and control, regarding the level of serum magnesium (p>0.05). Table 3 shows frequency of serum level of magnesium and potassium in case and control group. The relationship of serum magnesium and potassium level with duration of medication use is shown in table 4.

**Table 3:** Frequency of serum level of magnesium and potassium in case and control groups

**Parameters**	**Case**	**Control**
**Serum magnesium level**		
Hypomagnesemia (<1.7 mg/dl) Normal (1.7-2.6 mg/dl) Hypermagnesemia (>2.6 mg/dl)	16 (12.1) 110(83.3) 6 (4.5)	18 (13.7) 105 ( 80.1) 8 ( 6.1)
**Serum potassium level**		
Hypokalemia(<3.5 mEq/L) Normal(3.5-5 mEq/L) Hyperkalemia (>5 mEq/L)	29 (22) 103 (78) 0	4 (3.1) 122 (93.1) 5 (3.8)

**Table 4:** The relationship of serum magnesium and potassium level with duration of medication use

**Elements**	**Duration of medication use (year)**	**p-value**
Hypomagnesemia (<1.7 mg/dl) Normal (1.7-2.6 mg/dl) Hypermagnesemia (>2.6 mg/dl)	1.43± 2.19 1.51± 2.49 1.78± 2.99	0.903
Hypokalemia(<3.5 mEq/L) Normal(3.5-5 mEq/L) Hyperkalemia (>5 mEq/L)	2.85± 1.92 1.35±2.5	<0.001

* One way anova

As demonstrated in table 4, there was a significant relationship between serum levels of potassium and duration of medication use (p<0.01). The mean age of patients in hypomagnesemia (<1.7 mg/dl), normal (1.7-2.6 mg/dl) and hypermagnesemia groups (>2.6 mg/dl) was 56.4±14.4, 52.2±16.6 and 45.4±10.2, respectively. In addition the mean age of patients in hypokalemia (<3.5 mEq/L), normal (3.5-5 mEq/L) and hyperkalemia groups (>5 mEq/L) was 49.2±10.3, 53±16.8 and 45.4±18.4, respectively. The relationship between serum level of elements including Mg and K with age groups showed that there was no significant relation between Mg and K level and age groups (P=0.09). Moreover, no relation was seen between age and elements such as potassium level (P=0.29). Frequency comparison of two groups (male and female groups), regarding serum level of magnesium and potassium showed that there was no significant difference between frequency of patients in two groups, regarding magnesium (P=0.218) and potassium (P=0.175).

Serum level of potassium in *omeprazole, *pantoprazole and other drug groups was 1.99±0.35, 2.07±0.31 and 2.30±0.68, respectively. In addition, the mean level of potassium in 3 groups was 3.94±0.54, 3.84±0.89, and 4.18±0.45, respectively. In addition, One way ANOVA test showed a significant difference between 3 groups, regarding serum level of potassium (P=0.001). However, no significant difference was seen between these groups* (omeprazole, *pantoprazole and other drugs groups), regarding serum level of magnesium (P=0.226). 

## Discussion

PPIs are a mainstay treatment for gastric acid related diseases (32). There is clinical concern regarding PPI-induced hypomagnesaemia as a consequence of long-term PPI use. PPI induced hypomagnesaemia leads to symptoms including convulsions, cardiac arrhythmia tetany, seizures, and convulsion. It also exposes patients at risk for secondary electrolyte disturbances like hypocalcemia (32). However, drug-induced hypomagnesemia is not unique to PPI. Other classes of drugs including diuretics, gentamycin (33) platinum-based cytostatic, calcineurin inhibitors, epidermal growth factor receptor (EGF-R) targeting drugs have been associated with hypomagnesemia (34, 35). 

In our study, no significant difference was observed between case and control, regarding the levels of serum magnesium. It indicates that the use of proton pump inhibitor (PPI) did not affect the level of serum magnesium compared to control group. 

The role of studies about the use of PPIs in hypomagnesemia is a controversy. Koulouridis et al., reported that the use of PPI was not associated with hypomagnesemia during admission in the hospitals (30). Chowdhry  et al., evaluated the role of PPIs on 2400 patients with hypomagnesemia and reported that no significant difference was seen between the mean level of magnesium in PPI users and nonusers. The findings of two studies were consistent with our study. Moreover, according to these findings, taking PPIs in different doses, with or without concomitant diuretics was not associated with hypomagnesemia. Therefore, it seems that routine screening of serum magnesium in PPI may be unnecessary (36). 

Dunkin et al., revealed the lower level of serum Mg level in patients receiving PPI therapy than those not receiving PPIs (31). These findings imply that long-term use of PPIs was associated with Mg deficiency and insufficiency status. The mechanism that illustrates the cause of lower serum level of Mg in patients receiving PPIs is not clear (31). They reported that one limitation of this study was that data of serum magnesium level before treatment in these patients were unavailable; which was consistent with our study. 

Douwes et al., reported that the use of PPIs in kidney transplant recipients was associated with the risk of hypomagnesemia. They reported that the plasma level of magnesium was lower in PPIs users, indicating that hypomagnesemia was caused by impaired gastrointestinal absorption compared to renal magnesium wasting (37). It is assumed that PPIs inhibit the absorption of active magnesium via TRPM 6 and 7 channels in the intestine (37). Cheungpasitporn et al., reported that the use of PPIs was associated with hypomagnesemia and cardiovascular events. Moreover, they believed that physicians should consider the patients who are taking PPIs are at risk for cardiovascular events from hypomagnesemia, particularly those who have impairment in gastrointestinal absorptive capacity for magnesium and renal losses of magnesium due to diuretics or poor nutrition (38). Hess et al., in another study reported hypomagnesemia in 13% of PPIs users. They reported that single nucleotide polymorphism in TRPM6 (rs3750425 and rs2274924) increases the risk for PPI-induced hypomagnesemia approximately 5.8-fold (39). Therefore, according to this finding, single nucleotide polymorphism of some genes can increase the risk for PPI-induced hypomagnesemia. Luck et al., reported that the patient’s age is an influential factor on hypomagnesemia and older patients had a higher risk of developing hypomagnesemia (26). Therefore, the development of hypomagnesemia in patients using PPIs is influenced by several factors. In our study, 70 % of females using PPIs had hypomagnesemia, whereas 29 % of males had hypomagnesemia. But no significant relation was seen between serum magnesium level with age and sex. Dunkin et al., reported that although female PPIs users had lower serum level of Mg than males, the mean serum magnesium level was independent of gender and age, which was consistent with our study. The cause of lower level of serum Mg in women compared to men was unclear (31). In addition, no significant difference was seen between serum levels of magnesium, regarding the type of proton pump inhibitor such as omeprazole, pantoprazole and other drugs. It appears that the type of PPIs did not affect the serum level of magnesium. Dunkin et al., reported that mean serum level of Mg in esomeprazole users was higher than those using omeprazole or pantoprazole, whereas no significant difference was observed between these groups, which was consistent with our study (31).

In the current study, the mean level of potassium in case group (3.92±0.64) was significantly lower than the control group (4.20±0.43). Maede et al., revealed that the use of omeprazole decreased serum potassium level via accelerating urinary potassium excretion. Our findings were consistent with Maede’s study, regarding the serum level of potassium (40). Wainwright et al., reported that though some potassium is lost from the stomach, renal excretion of potassium is the primary cause of hypokalemia. They also revealed that vomiting can lead to hypokalemia via two pathways. First; gastric acid loss leads to hypochloraemic metabolic alkalosis which increases filtered bicarbonate load in the nephron and subsequently increases distal sodium bicarbonate delivery. Secondly, hypovolemia causes activation of the renin-angiotensin-aldosterone axis. Standard treatment of severe hypokalemia in patients with bulimia nervosa, anorexia and persistent self-induced vomiting includes intravenous replacement of potassium and correction of hypovolemia (41). In addition, the significant difference was seen between serum levels of potassium, regarding the type of proton pump inhibitor and duration of medication use. At the time of PPI administration, it seems necessary to pay attention to the duration of medication use and the type of proton pump inhibitor.

In conclusion, based on these results, long-term use of proton pump inhibitors is not associated with hypomagnesemia in GERD patients. However, long-term use of PPIs may reduce serum potassium levels in these patients. Therefore, the periodic evaluation of serum Mg level in PPI-treated patients seems to be unnecessary. Furthermore, more studies should be done regarding the level of potassium after the long-term use of proton pump inhibitor.

## Funding:

None

## Conflict of Interest:

There is no conflict of interest.
